# Medical Diagnosis with Special Reference to Practical Medicine

**Published:** 1884-06

**Authors:** 


**Medical Diagnosis with Special Reference to Practical Medicine.** A guide to the knowledge and discrimination of diseases. By I. M. Da Casta, M. D., LL. D., Prof. of Practice of Medicine at the Jefferson Medical College, Philadelphia. Illustrated with engravings on wood. Sixth edition, revised. Philadelphia: J. B. Lippincott & Co. 1884.

It is hardly necessary to do more than announce a new edition of this admirable work. A book so wtdely known and so highly valued as to have attained, in a few years, to its sixth edition, needs no praise. Like a good many other books of American authorship, it is extensively used in England, and has been translated into German, French and Spanish. This new edition contains a good deal of new matter and a number of wood cuts have been added. It is one of the books which ought to be in every physician's library.

